# Associations of falls and severe falls with blood pressure and frailty among Chinese community-dwelling oldest olds: The Chinese Longitudinal Health and Longevity Study

**DOI:** 10.18632/aging.203174

**Published:** 2021-06-23

**Authors:** Yujian Song, Yujiao Deng, Jianhua Li, Benchuan Hao, Yulun Cai, Jianqiao Chen, Haiyan Shi, Weihao Xu

**Affiliations:** 1 Graduate School of Medical School of Chinese PLA, Beijing 100853, China; 2 Department of Ultrasound, The First Medical Center of Chinese PLA General Hospital, Beijing 100853, China; 3 Department of Geriatric Cardiology, The Second Medical Center & National Clinical Research Center for Geriatric Diseases, Chinese PLA General Hospital, Beijing 100853, China; 4 Department of Gastroenterology, The Second Medical Center & National Clinical Research Center for Geriatric Diseases, Chinese PLA General Hospital, Beijing 100853, China; 5 Haikou Cadre's Sanitarium of Hainan Military Region, Haikou 570203, China

**Keywords:** frailty, blood pressure, falls, Chinese, oldest old

## Abstract

Introduction: Falls are a leading cause of death among Chinese oldest olds. However, studies on Chinese community-dwelling older adults are lacking. We aimed to identify the associations of falls and severe falls with blood pressure and frailty among Chinese community-dwelling oldest olds.

Methods: Cross-sectional analyses were conducted with 6,595 community-dwelling oldest olds (aged ≥80 years) from 22 Chinese provinces from the Chinese Longitudinal Health and Longevity Study (CLHLS). Systolic BP (SBP) and diastolic BP (DBP) were measured twice at participants’ homes, and a 38-item frailty index was used to assess the frailty status of participants. Falls and severe falls were confirmed through face-to-face interviews. Multivariate logistic regression was used to investigate the associations of BP and frailty with falls and severe falls.

Results: The mean participant age was 91.0 years, and 56.1% were female. In total, 24.2% participants had a history of fall and 8.3% had a history of severe falls. The multivariate-adjusted odds ratio (OR) for falls among the oldest old with SBP ≥140 mm Hg compared to those with an SBP of 120–129 mm Hg was 1.20 (95% confidence interval [CI], 1.01–1.44). The adjusted OR for falls among frail participants compared to robust participants was 1.39 (95% CI, 1.02–1.89). DBP and pre-frailty were not associated with falls after multivariate adjustment. SBP, DBP, and frailty status were not associated with severe falls after multivariate adjustment.

Conclusions: SBP and frailty but not DBP and pre-frailty are associated with increased odds of falls among Chinese community-dwelling oldest olds.

## INTRODUCTION

Falls are a leading cause of hospitalization, disability, and even death among older adults [1, 2], particularly the oldest olds [3]. Falls are the leading cause of deaths due to unintentional injury worldwide [4]. In China, falls are the leading cause of injury-related deaths among older adults [5]. Approximately 1 in 3 adults aged ≥65 years and 50% of those aged ≥80 years experienced ≥1 falls every year [6, 7]. Estimated annual medical costs of falls-related fatal or nonfatal injuries are $50 billion [7, 8]. Considering the substantial social, economic, and health burdens, identifying modifiable risk factors for falls is crucial.

Studies have reported the association between orthostatic hypotension and the increased risk of falls among outpatients and nursing home residents [9, 10]. However, only few studies on the effects of conventionally measured blood pressure (BP) on falls among community-dwelling older adults exist [11, 12]. Although studies have demonstrated that a low BP can increase mortality risk, the association between BP and falls remains unclear [13–15], with inconsistent results showing a nonlinear association between BP and the incidence of falls [11, 16].

Frailty is a complex and universal aging-related condition characterized by physiological, psychological, and social deficits in older adults [17, 18]. Studies from high-income countries have confirmed the association between frailty and falls [19–22]. However, studies from low- and middle-income countries are scarce [18].

China accounts for one-fifth of the world’s oldest old population, with the largest and fastest-growing aging population globally [23]. The oldest old population in China is projected to reach 150 million by 2050 [17, 24]. This growing population of the oldest old in China can impose a severe burden on the healthcare system. Identifying the associations of BP and frailty with falls and severe falls would help identify individuals at a high risk of falls and administer early interventions to avoid fall-related injury or mortality.

We used the data from the Chinese Longitudinal Health and Longevity Study (CLHLS) to identify the associations of BP and frailty with falls and severe falls among the community-dwelling oldest old. CLHLS is a population-based study, and findings from this study would be crucial in developing interventions for prevention of falls among the oldest old.

## MATERIALS AND METHODS

### Study population

We used the data from CLHLS, an ongoing, prospective cohort study of Chinese community-dwelling older adults. CLHLS began in 1998 with the aim to identify the determinants of longevity and comprises a nationally representative sample of older adults from 22 of 34 Chinese provinces. Follow-up interviews were conducted every 2 years before the third wave (2000 and 2002), and then every 3 years after the third wave (2005, 2008, 2011, 2014, and 2017–2018). The study added individuals (adults aged ≥80 years in the second wave and adults aged ≥65 years in the third and subsequent waves) to compensate for participants who died or were lost to follow-up. Demographic information, personality, emotional status, general ability, lifestyle, activities of daily living, and physical health were collected by trained investigators using an extensive questionnaire during in-home face-to-face interviews. The quality of data in CLHLS has been systematically assessed for the accuracy of age reporting, attrition randomness, reliability, validity, and consistency using numerous measures [25, 26].

This study used the data from the 2017–2018 wave. We included 15,874 participants from CLHLS. We then excluded 5,455 participants aged <80 years. We further excluded 3,607 participants lacking sufficient frailty index (FI) item responses, 61 lacking BP values, and 156 without falls records. In total, 6,595 participants were included in the analyses (Supplementary Figure 1).

### Blood pressure

A mercury sphygmomanometer was used to measure BP while participants were in a seated position in their home. Two measurements were taken and the average of the two values was used for analysis. SBP and DBP were recoded as categorical variables with SBP (<100, 110–119, 120–129, 130–139, and ≥140 mm Hg) and DBP (<60, 60–69, 70–79, 80–89, and ≥90 mm Hg). Reference groups for SBP and DBP were 120–129 mm Hg and 70–79 mm Hg, respectively.

### Frailty index

Frailty status was assessed using a 38-item FI. We constructed the FI following a standard procedure [27]. The FI counts deficits in health. Health deficits were defined as symptoms, signs, disabilities, and diseases [27]. Criteria for health deficits to be included in the FI were: association with the health status; a prevalence >1% increasing with age; no early-age onset; and affecting several physiological systems. Each health deficit was scored as 0 (absence), 1 (presence), or missing. For each participant, the FI score was calculated as the sum of deficit scores divided by the number of deficits included and ranged from 0 to 1. We constructed a 38-item FI following an established study using data from CLHLS [28]. We included participants with ≥30 items. After the FI was calculated, all participants were categorized as robust (FI ≤0.12), pre-frail (0.12< FI ≤0.25), or frail (FI >0.25) [29]. Variables used to construct the FI and coding are defined in Supplementary Table 1.

### Falls and severe falls

A history of fall or severe fall was established using the questionnaire based on self- or kin-reporting. A fall was defined as an accidental event that caused the participant to unintentionally fall to the floor or other lower levels. A severe fall was defined as a fall that caused significant injury requiring medical treatment. Occurrence of falls was ascertained by the question “Have you fallen down in the last year?” (Yes/No).

### Covariates

We adjusted for several factors in the logistic model including age (80–89, 90–99, and 100+ years), sex, current smoking and drinking status, marital status (married/living together, widowed, and others), education years (≥11 and <11), and body mass index (BMI [kg/m^2^], underweight [<18.5], normal [18.5–24.9], overweight [25.0–29 .9], obese ≥30]) [30].

### Statistical analyses

Quantitative variables were described as means ± standard deviations, and qualitative variables were reported as absolute proportions (%). Binary logistic regression models were used to calculate the odds ratio (OR) and 95% confidence interval (CI) for falls and severe falls associated with BP level and FI. We included age and sex in model 1. We further included education years, marital status, current smoking status, current drinking status, and BMI in model 2. A third model evaluating the associations of SBP and DBP with falls and severe falls included the FI. We also performed sensitivity analyses by calculating the E-value to assess the effect of potential unmeasured confounding on the observed associations between BP, frailty and falls [31]. Previous studies have shown the U/J shaped association between BP and adverse outcomes, including mortality and cardiovascular outcomes [32, 33]. Thus, we hypothesize that the association between SBP, DBP and falls might be non-linear, and used two methods (linear and quadratic terms) to assess trends across levels of SBP and DBP. We repeated the analyses for the associations of SBP and of DBP with falls and severe falls for participants with different frailty status.

All analyses were performed using SPSS 25.0 for Windows (SPSS Inc., Chicago, IL). The statistical tests were 2-tailed, and *P* < 0.05 was considered statistically significant.

### Statement of ethics

The paper is exempt from ethical committee approval since the study was a retrospective, anonymized analysis.

## RESULTS

### Participant characteristics

The final analyses covered 6,595 participants, including 45.4% octogenarians, 34.0% nonagenarians, and 20.7% centenarians. The mean age was 91.0 ± 7.5 years, and 56.1% were female. Of total, 24.2% had a history of a fall in the previous year, and 8.3% had a history of a severe fall; 35.3% were frail, 59.2% were pre-frail, and 5.5% were robust. Frail participants were older and more likely to be females, current smokers, current drinkers, and underweight. Frail participants were less likely to be currently married and living with their spouse (Table 1). Population characteristics by SBP and DBP levels are summarized in Supplementary Tables 2 and 3. Baseline characteristics of included and excluded participants were shown in Supplementary Table 4. Excluded participants were older and had higher proportion of obesity and normal weight than those included.

**Table 1:** Characteristics of CLHLS (Chinese Longitudinal Healthy Longevity Survey) participants ≥ 80 years of age by frail status.

**Characteristic**	**Frail status**		
	**Robust (*n* = 361)**	**Pre-frail (*n* = 3,904)**	**Frail (*n* = 2,330)**
^***^Age, years, mean (SD)	89.2 (7.3)	90.4 (7.4)	92.2 (7.5)
^***^Female, [*n* (%)]	188 (52.1)	2,120 (54.3)	1,396 (59.9)
^***^Marital status Currently married and living with spouse, [*n* (%)]	103 (28.5)	1,089 (27.9)	508 (21.8)
Education years, < 11 years, [*n* (%)]	259 (71.7)	3057 (78.3)	1,936 (83.1)
^***^Current smoker, [*n* (%)]	68 (18.8)	566 (14.5)	252 (10.8)
^***^Current drinker, [*n* (%)]	69 (19.1)	554 (14.2)	247 (10.6)
SBP, mmHg, mean (SD)	141.3 (21.5)	140.7 (21.7)	139.7 (21.4)
DBP, mmHg, mean (SD)	79.2 (10.8)	78.9 (11.5)	78.6 (11.6)
^***^BMI categories, [*n* (%)]			
Underweight	81 (22.4)	842 (21.6)	601 (25.8)
Normal	222 (61.5)	2,372 (60.8)	1,290 (54.9)
Overweight	48 (13.3)	550 (14.1)	325 (13.9)
Obesity	10 (2.8)	140 (3.6)	114 (4.9)
^***^History of Falls, [*n* (%)]	80 (22.2)	858 (22.0)	655 (28.1)
History of Severe falls, [*n* (%)]	28 (7.8)	286 (7.3)	236 (10.1)

Abbreviations: SD: Standard Deviation; SBP: Systolic Blood Pressure; DBP: Diastolic Blood Pressure; BMI: Body mass index.

^***^P < 0.001

### Associations of SBP and DBP with falls and severe falls

After multivariate adjustment, only SBP ≥140 mm Hg was associated with an increased OR for falls (1.20; 95% CI, 1.01–1.44). Other SBP levels and all DBP levels were not associated with falls. In a multivariate model, SBP and DBP were not associated with severe falls. No linear or quadratic trends in ORs for falls and severe falls across SBP and DBP levels were seen, which indicated that the association was not the higher the SBP and DBP levels, the higher odds for falls and severe falls; or both lower and higher SBP and DBP levels were associated with higher odds for falls and severe falls (Tables 2 and 3).

**Table 2:** Odds ratio for falls and severe falls by systolic blood pressure.

**Outcome**	**Systolic blood pressure, mmHg**					***p*-trend**	
	**<110**	**110–119**	**120–129**	**130–139**	**≥140**	**linear**	**quadratic**
**Falls**	**OR (95% CI)**						
Model 1	0.97 (0.72–1.31)	1.14 (0.90–1.44)	1.00 (ref)	1.11 (0.92–1.33)	1.08 (0.92–1.26)	0.67	0.71
Model 2	1.16 (0.84–1.60)	1.21 (0.94–1.58)	1.00 (ref)	1.17 (0.96–1.42)	1.20 (1.00–1.43)	0.36	0.40
Model 3	1.17 (0.85–1.62)	1.23 (0.95–1.59)	1.00 (ref)	1.17 (0.85–1.62)	1.20 (1.01–1.44)	0.35	0.35
**Severe falls**	**OR (95% CI)**						
Model 1	1.09 (0.71–1.67)	0.86 (0.59–1.25)	1.00 (ref)	1.01 (0.77–1.33)	0.91 (0.71–1.16)	0.44	0.76
Model 2	1.18 (0.75–1.89)	0.84 (0.55–1.28)	1.00 (ref)	1.04 (0.77–1.41)	1.04 (0.79–1.36)	0.78	0.67
Model 3	1.20 (0.75–1.90)	0.85 (0.56–1.30)	1.00 (ref)	1.04 (0.77–1.41)	1.04 (0.80–1.37)	0.77	0.63

Abbreviations: OR: Odds ratio; CI: Confidence interval.

Model 1 includes adjustment for age, sex.

Model 2 includes variables in Model 1 and current smoking and drinking status, marital status, education years and body mass index.

Model 3 includes variables in Models 1 and 2 and frail status.

Linear *p*-trend represents the *p*-value for a linear trend across the systolic blood pressure categories. Quadratic *p*-trend represents the *p*-value for a deviation from linearity across the systolic blood pressure categories.

**Table 3:** Odds ratio for falls and severe falls by diastolic blood pressure.

**Outcome**	**Diastolic blood pressure, mmHg**					***p*-trend**	
	**<60**	**60–69**	**70–79**	**80–89**	**≥90**	**linear**	**quadratic**
**Falls**	**OR (95% CI)**						
Model 1	1.07 (0.76–1.50)	1.22 (1.02–1.45)	1.00 (ref)	1.01 (0.87–1.16)	0.96 (0.81–1.14)	0.05	0.64
Model 2	1.10 (0.76–1.58)	1.18 (0.97–1.43)	1.00 (ref)	1.04 (0.89–1.22)	0.98 (0.82–1.18)	0.22	0.73
Model 3	1.10 (0.76–1.58)	1.17 (0.97–1.43)	1.00 (ref)	1.04 (0.89–1.22)	0.98 (0.69–1.03)	0.22	0.74
**Severe falls**	**OR (95% CI)**						
Model 1	0.97 (0.57–1.64)	0.96 (0.72–1.27)	1.00 (ref)	0.93 (0.75–1.16)	0.94 (0.73–1.22)	0.75	0.88
Model 2	1.09 (0.63–1.88)	0.95 (0.70–1.29)	1.00 (ref)	0.97 (0.76–1.23)	1.03 (0.78–1.37)	0.85	0.69
Model 3	1.08 (0.62–1.87)	0.95 (0.69–1.29)	1.00 (ref)	0.96 (0.76–1.23)	1.04 (0.78–1.37)	0.84	0.69

Abbreviations: OR: Odds ratio; CI: Confidence interval.

Model 1 includes adjustment for age, sex.

Model 2 includes variables in Model 1 and current smoking and drinking status, marital status, education years and body mass index.

Model 3 includes variables in Models 1 and 2 and frail status.

Linear *p*-trend represents the *p*-value for a linear trend across the diastolic blood pressure categories. Quadratic *p*-trend represents the *p*-value for a deviation from linearity across the diastolic blood pressure categories.

### Association between frailty status and falls and severe falls

The multivariate-adjusted ORs for falls among participants who were pre-frail and frail vs robust were 1.06 (95% CI, 0.78–1.43) and 1.39 (95% CI, 1.02–1.89), respectively. After multivariate adjustment, pre-frailty (OR, 0.98; 95% CI, 0.61–1.59) and frailty (OR, 1.30; 95% CI, 0.80–2.11) were not associated with severe falls (Table 4).

**Table 4:** Odds ratio for falls and severe falls by frailty status.

**Outcome**	**Frailty index**		
	**Robust**	**Pre-frail**	**Frail**
**Falls**	**OR (95% CI)**		
Model 1	1.00 (ref)	0.97 (0.75–1.26)	1.31 (1.00–1.70)
Model 2	1.00 (ref)	1.05 (0.78–1.42)	1.37 (1.01–1.87)
Model 3	1.00 (ref)	1.06 (0.78–1.43)	1.39 (1.02–1.89)
**Severe falls**	**OR (95% CI)**		
Model 1	1.00 (ref)	0.91 (0.61–1.37)	1.25 (0.83–1.88)
Model 2	1.00 (ref)	0.99 (0.62–1.60)	1.31 (0.81–2.12)
Model 3	1.00 (ref)	0.98 (0.61–1.59)	1.30 (0.80–2.11)

Abbreviations: OR: Odds ratio; CI: Confidence interval.

Model 1 includes adjustment for age, sex.

Model 2 includes variables in Model 1 and current smoking and drinking status, marital status, education years and body mass index.

Model 3 includes variables in Models 1 and 2 and systolic blood pressure and diastolic blood pressure.

### Subgroup analysis

After multivariate adjustment, only SBP > 140 mm Hg (OR, 1.62; 95% CI, 1.22–2.16) and SBP of 130–139 mm Hg (OR, 1.56; 95% CI, 1.13–2.13) were associated with falls among frail participants. SBP and DBP were not associated with severe falls among frail or non-frail participants (Supplementary Tables 5 and 6).

### Sensitivity analysis

The E value (CI bound) was 1.69 (1.11) for the association between SBP (>140 mmHg) and falls, and 2.13 (1.16) for the association between frailty and falls. Considering that we have controlled for most important confounders, an unmeasured confounder with such a strong association with falls is less likely to be missed.

## DISCUSSION

In our study of the Chinese community-dwelling oldest-old, only SBP >140 mm Hg was associated with increased odds for falls after multivariate adjustment and other levels of SBP and DBP were not associated with falls. In addition, frailty assessed using FI was associated with substantially increased odds for falls. However, SBP, DBP, and frailty status were not associated with severe falls after multivariate adjustment.

Previous epidemiological studies exploring the association between BP and falls among older adults have shown conflicting results [11, 12, 16]. An Australian study of 3,544 community-dwelling adults aged ≥60 years showed that SBP ≥140 mm Hg vs. SBP <140 mm Hg and DBP ≥90 mm Hg vs. DBP <90 mmHg were both associated with lower odds for falls among women but not men. Furthermore, the odds for falls among men were lower but not for women with SBP <120 mm Hg vs an SBP of 120–139 mm Hg and DBP <80 mm Hg vs a DBP of 80 89 mm Hg [12]. Banach et al. used the data from the REGARDS study and found that no association between SBP and recurrent falls across 3 age groups (55–64, 65–74, and ≥75 years) in the fully adjusted model [16]. Another study by Bromfield et al. showed that SBP and DBP were not associated with a risk for serious fall injuries after multivariate adjustment [11]. The findings of our study are inconsistent with the results of the aforementioned studies. The inconsistencies could be explained by several reasons. First, the age of the study population was not the same across studies. Second, different BP subgroups and reference groups might also contribute to the discrepancies. Third, study design, adjustment factors, and study endpoints varied across different studies. There are several plausible explanations for the association between high blood pressure and falls. First, patients who have hypertension or high blood pressure also have higher odds for sarcopenia which is one of the most significant risk factors for falls, especially in Asian population [34]. Second, hypertension is an independent risk factor for cardiovascular disease, thus hypertensive patients often comorbid with coronary heart disease or cerebrovascular disease [35, 36]. As a result, multimorbidity and polypharmacy, which are well-recognized risk factors for falls, are very common in older patients with hypertension [37]. Third, patients with hypertension are at increased risk for cognitive dysfunction, which is also a significant risk factor for falls.

Several studies have reported the association between frailty or indicators of frailty and a higher risk of falls among older adults [38–42]. The meta-analysis by Deandrea et al. found that cognitive impairment (OR, 1.36; 95% CI, 1.12–1.65), depression (OR, 1.63; 95% CI, 1.36–1.94), physical activity (OR, 1.20; 95% CI, 1.04–1.38), and gait disturbance (OR, 2.06; 95% CI, 1.82–2.33) were each associated with increased likelihood of falls [43]. The meta-analyses by Cheng et al. and Fhon et al. both showed that frailty was a risk factor for falls (OR, 2.50; 95% CI, 1.58–3.96 and OR, 1.82; 95% CI, 1.50–2.13, respectively) [44, 45]. In addition, Cheng et al. also found that frailty was associated with a higher risk of recurrent falls (OR, 2.77; 95% CI, 2.06–3.72) [45]. Of note, few of the studies included in the aforementioned meta-analyses used FI to assess frailty status. Studies focusing on the oldest old population are lacking. Thus, our finding that frailty assessed using an FI is associated with falls in the oldest old population has potential implications for future research and practice.

The present study has several strengths. First, to our knowledge, this is the first study on the associations of BP and frailty with falls and severe falls among the oldest olds. Second, our study sample is large and nationally representative. Third, BP was measured following a standardized protocol, and frailty status was assessed using a 38-item FI that has been well-established in the CLHLS study [28].

This study also has several limitations. First, the cross-sectional design precluded determining the causal associations between BP and frailty and falls. Future research on CLHLS participants with a prospective design is needed to substantiate this association. Second, both falls and severe falls were ascertained based on self-reports by the participants or their proxies, which might lead to omission of study outcomes. Consequently, the prevalence of falls and severe falls might have been underestimated. Third, although the FI has been validated in various populations, it is difficult to construct and inconvenient to use in clinical practice. Fourth, although a wide range of confounders were included in the logistic analysis, residual confounding could still exist. However, the E value of our results indicated that an unmeasured confounder was less likely to change the association. Fifth, BP data used in this study was the average of 2 BP measurements in the same time period, which could not reflect the true blood pressure status of the research subjects. Future studies applied with ambulatory blood pressure monitoring (ABPM) for BP measurement were warranted to further examine the association between BP and falls. Moreover, our findings may not be generalizable to those aged 65–79 years and to older adults residing in nursing homes.

## CONCLUSIONS

Chinese community-dwelling oldest old with an SBP ≥140 mm Hg and FI >0.25 are at an increased risk of falls. Other SBP and DBP levels and 0.12 < FI ≤0.25 were not associated with falls. SBP, DBP, and frailty status are not independently associated with the risk for severe falls. These findings indicate that SBP ≥140 mm Hg might increase the risk of falls among the oldest old. In addition, frailty might be a risk factor for falls among the oldest old; thus, assessment of frailty should be considered to identify those at a high risk of falls.

## Supplementary Materials

Supplementary Figure 1

Supplementary Tables
