# Supplementary material for: Associations of falls and severe falls with blood pressure and frailty among Chinese community-dwelling oldest olds: The Chinese Longitudinal Health and Longevity Study
Source: Aging (Albany NY). 2021 Jun 23;13(12):16527–40. doi: 10.18632/aging.203174 (PMC8266320; doi:10.18632/aging.203174)
Supplement: Supplementary Figure 1 [file aging-13-203174-s001.pdf]

SUPPLEMENTARY FIGURE

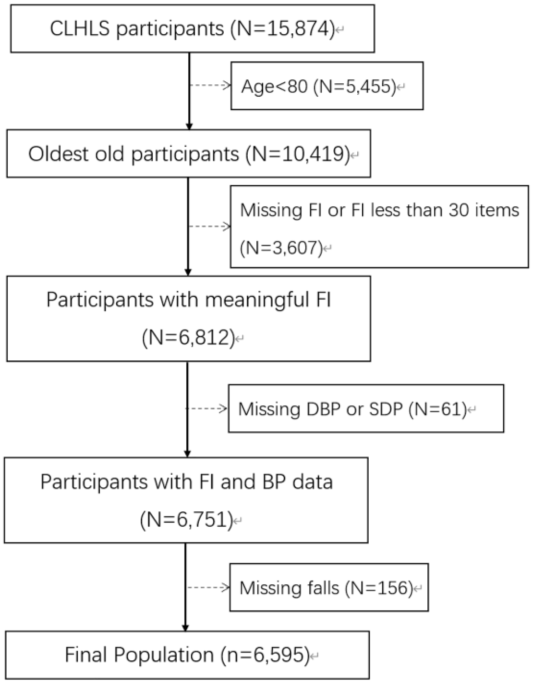

Supplementary Figure 1. Exclusion criteria for CLHLS analysis linked falls analysis.
