# Supplementary material for: Associations of falls and severe falls with blood pressure and frailty among Chinese community-dwelling oldest olds: The Chinese Longitudinal Health and Longevity Study
Source: Aging (Albany NY). 2021 Jun 23;13(12):16527–40. doi: 10.18632/aging.203174 (PMC8266320; doi:10.18632/aging.203174)
Supplement: Supplementary Tables [file aging-13-203174-s002.pdf]

SUPPLEMENTARY TABLES

Supplementary Table 1. Variables used to construct the frailty index.

	Variables	Data Type	Cut-off point
1	Self-reported health	Ordinal	V. good = 0, good = 0.25; so so = 0.5, bad = 0.75, very bad = 1
2	Feel fearful or anxious	Ordinal	Always = 1, often = 0.75, sometimes = 0.5, seldom = 0.25, rarely or never = 0
3	Feel useless with age	Ordinal	Always = 1, often = 0.75, sometimes = 0.5, seldom = 0.25, rarely or never = 0
4	Bathing	Ordinal	Without assistance = 0, one part assistance = 0.5, more than one part assistance = 1
5	Dressing	Ordinal	Without assistance = 0, one part assistance = 0.5, more than one part assistance = 1
6	Toileting	Ordinal	Without assistance = 0, one part assistance = 0.5, more than one part assistance = 1
7	Transferring	Ordinal	Without assistance = 0, one part assistance = 0.5, more than one part assistance = 1
8	Continence	Ordinal	Without assistance = 0, one part assistance = 0.5, more than one part assistance = 1
9	Feeding	Ordinal	Without assistance = 0, one part assistance = 0.5, more than one part assistance = 1
10	Visual function	Ordinal	Can see and distinguish = 0, can see only = 0.5, can't see = 1, blind = 1
11	Rhythm of heart	Binary	> = 80bpm = 1; <80bpm = 0
12	Hand behind neck	Ordinal	Both hands = 0, left hand = 0.5, right hand = 0.5, neither hand = 1
13	Hand behind lower back	Ordinal	Both hands = 0, left hand = 0.5, right hand = 0.5, neither hand = 1
14	Able to stand up from sitting	Ordinal	Yes, without using hands = 0, Yes, using hands = 0.5, no = 1
15	Able to pick up a book from the floor	Ordinal	Yes, standing = 0, Yes, sitting = 0.5, no = 1
16	Number of times suffering from serious illness in the past two years	Ordinal	Yes = 2, no = 0
17	Hypertension	Binary	Yes = 1, no = 0
18	Diabetes	Binary	Yes = 1, no = 0
19	Heart disease	Binary	Yes = 1, no = 0
20	Stroke or CVD	Binary	Yes = 1, no = 0
21	Bronchitis, emphysema, pneumonia, asthma	Binary	Yes = 1, no = 0
22	Tuberculosis	Binary	Yes = 1, no = 0
23	Cancer	Binary	Yes = 1, no = 0
24	Gastric or duodenal ulcer	Binary	Yes = 1, no = 0
25	Parkinson	Binary	Yes = 1, no = 0
26	Bedsore	Binary	Yes = 1, no = 0
27	Able to hear	Binary	Yes = 1, no = 0
28	Interviewer rated health	Ordinal	Surprisingly healthy = 0, relatively healthy = 0, moderately ill = 0.5, very ill = 1
29	Look on the bright side of things	Ordinal	Always = 0, often = 0.25, sometimes = 0.5, seldom = 0.75, rarely or never = 1
30	Keep my belongings neat and clean	Ordinal	Always = 0, often = 0.25, sometimes = 0.5, seldom = 0.75, rarely or never = 1
31	Make own decisions	Ordinal	Always = 0, often = 0.25, sometimes = 0.5, seldom = 0.75, rarely or never = 1
32	Housework at present	Ordinal	Almost everyday = 0, not daily, but once for a week = 0.25, not weekly, but at least once for a month = 0.5, not monthly, but sometimes = 0.75, never = 1
33	Able to use chopsticks to eat	Ordinal	Yes = 1, no = 0
34	Number of steps used to turn around a 360 degree turn without help	Interval	> = 6 steps = 1, <6 steps = 0
35	Cataract	Binary	Yes = 1, no = 0
36	Glaucoma	Binary	Yes = 1, no = 0
37	Other chronic disease	Categorical	Yes = 1, no = 0
38	Prostate Tumor	Binary	Yes = 1, no = 0

Supplementary Table 2. Characteristics of CLHLS (Chinese Longitudinal Healthy Longevity Survey) participants ≥ 80 years of age by systolic blood pressure.

Characteristic	Systolic Blood Pressure, mmHg				
	**<110 (*n* = 316)**	**110–119 (*n* = 557)**	**120–129 (*n* = 1,133)**	**130–139 (*n* = 1,510)**	**≥140 (*n* = 3,079)**
***Age, years, mean (SD)	93.0 (7.7)	91.4 (7.4)	91.3 (7.5)	90.3 (7.4)	90.8 (7.5)
***Female, [*n* (%)]	179 (56.6)	297 (53.3)	616 (54.4)	799 (52.9)	1,810 (58.8)
*Marital status Currently married and living with spouse, [*n* (%)]	73 (23.1)	148 (26.6)	272 (24.0)	439 (29.1)	767 (24.9)
**Education years, <11 years, [*n* (%)]	253 (80.1)	448 (80.4)	917 (80.9)	1,237 (81.9)	2,395 (77.8)
***Current smoker, [*n* (%)]	60 (19.0)	90 (16.2)	157 (13.9)	211 (14.0)	366 (11.9)
*Current drinker, [*n* (%)]	42 (13.3)	76 (13.6)	144 (12.7)	239 (15.8)	369 (12.0)
***SBP, mmHg, mean (SD)	101.1 (7.2)	114.5 (3.1)	124.4 (3.1)	134.1 (3.1)	158.1 (16.6)
***DBP, mmHg, mean (SD)	64.0 (7.9)	70.2 (7.8)	74.5 (7.9)	77.7 (8.6)	84.1 (11.6)
***BMI categories, [*n* (%)]					
Underweight	138 (43.7)	170 (30.5)	294 (25.9)	317 (21.0)	605 (19.6)
Normal	152 (48.1)	324 (58.2)	655 (57.8)	917 (60.7)	1,836 (59.6)
Overweight	16 (5.1)	47 (8.4)	141 (12.4)	198 (13.1)	521 (16.9)
Obesity	10 (3.2)	16 (2.9)	43 (3.8)	78 (5.2)	117 (3.8)
Frailty index, mean (SD)	0.2 (0.1)	0.2 (0.1)	0.2 (0.1)	0.2 (0.1)	0.2 (0.1)
History of Falls, [*n* (%)]	73 (23.1)	141 (25.3)	261 (23.0)	370 (24.5)	748 (24.3)
History of Severe falls, [*n* (%)]	31 (9.8)	42 (7.5)	99 (8.7)	130 (8.6)	249 (8.1)

Abbreviations: SD: Standard Deviation; SBP: Systolic Blood Pressure; DBP: Diastolic Blood Pressure; BMI: Body mass index.
*$P < 0.05$; **$P < 0.01$; ***$P < 0.001$

Supplementary Table 3. Characteristics of CLHLS (Chinese Longitudinal Healthy Longevity Survey) participants ≥ 80 years of age by diastolic blood pressure.

Characteristic	Diastolic Blood Pressure, mmHg				
	<60 (*n* = 196)	60–69 (*n* = 909)	70–79 (*n* = 2,040)	80–89 (*n* = 2,207)	≥90 (*n* = 1,243)
***Age, years, mean (SD)	93.3 (7.7)	91.9 (7.4)	90.6 (7.3)	90.9 (7.6)	90.6 (7.5)
**Female, [*n* (%)]	108 (55.1)	514 (56.5)	1100 (53.9)	1229 (55.7)	750 (60.3)
**Marital status<br>Currently married and living with spouse, [*n* (%)]	35 (17.9)	229 (25.2)	575 (28.2)	562 (25.5)	299 (24.1)
***Education years, <11 years, [*n* (%)]	158 (80.6)	733 (80.6)	1,613 (79.1)	1,775 (80.4)	972 (78.2)
*Current smoker, [*n* (%)]	40 (20.4)	138 (15.2)	270 (13.2)	280 (12.7)	156 (12.6)
Current drinker, [*n* (%)]	25 (12.8)	122 (13.4)	265 (13.0)	310 (14.0)	149 (12.0)
***SBP, mmHg, mean (SD)	119.8 (21.9)	126.2 (18.6)	134.7 (17.4)	141.9 (17.0)	160.6 (21.2)
***DBP, mmHg, mean (SD)	54.1 (4.4)	64.0 (2.8)	73.5 (3.0)	82.6 (2.8)	95.5 (7.5)
***BMI categories, [*n* (%)]					
Underweight	83 (42.3)	246 (27.1)	459 (22.5)	493 (22.3)	243 (19.5)
Normal	87 (44.4)	534 (58.7)	1,246 (61.1)	1,288 (58.4)	729 (58.7)
Overweight	17 (8.7)	100 (11.0)	264 (12.9)	328 (14.9)	214 (17.2)
Obesity	9 (4.6)	29 (3.2)	71 (3.5)	98 (4.4)	57 (4.6)
Frailty index, mean (SD)	0.2 (0.1)	0.2 (0.1)	0.2 (0.1)	0.2 (0.1)	0.2 (0.1)
History of Falls, [*n* (%)]	50 (25.5)	252 (27.7)	480 (23.5)	525 (23.8)	286 (23.0)
History of Severe falls, [*n* (%)]	17 (8.7)	77 (8.5)	174 (8.5)	179 (8.1)	103 (8.3)

Abbreviations: SD: Standard Deviation; SBP: Systolic Blood Pressure; DBP: Diastolic Blood Pressure; BMI: Body mass index.
*$P < 0.05$; **$P < 0.01$; ***$P < 0.001$

Supplementary Table 4. Characteristics of included and excluded participants of CLHLS (Chinese Longitudinal Healthy Longevity Survey) ≥ 80 years of age.

Characteristic	Included participants (*n* = 6,595)	Excluded participants (*n* = 3,824)
***Age, years, mean (SD)	91.0 (7.5)	94.7 (7.6)
Female, [*n* (%)]	3,701 (56.1)	2,210 (57.8)
Marital status<br>Currently married and living with spouse, [*n* (%)]	1,700 (25.8)	1,006 (26.3)
Education years, < 11 years, [*n* (%)]	5,269 (79.9)	3,136 (82.0)
Current smoker, [*n* (%)]	884 (13.4)	558 (14.6)
Current drinker, [*n* (%)]	871 (13.2)	493 (12.9)
SBP, mmHg, mean (SD)	140.5 (21.8)	138.1 (22.5)
DBP, mmHg, mean (SD)	78.8 (11.6)	77.8 (12.1)
***BMI categories, [*n* (%)]		
Underweight	1,524 (23.1)	812 (21.2)
Normal	3,884 (58.9)	2279 (59.6)
Overweight	923 (14.0)	507 (13.3)
Obesity	264 (4.0)	226 (5.9)

Abbreviations: SD: standard deviation; SBP: systolic blood pressure; DBP: diastolic blood pressure; BMI: body mass index.
***$P < 0.001$

Supplementary Table 5. Odds ratios for falls associated with systolic blood pressure and diastolic blood pressure, stratified by frail status.

Frail status*	Systolic blood pressure, mmHg					p-trend	
	<110	110–119	120–129	130–139	≥140	linear	quadratic
			OR** (95% CI)				
Non–frail	1.17 (0.78–1.75)	1.14 (0.82–1.57)	1.00 (ref)	0.96 (0.74–1.24)	0.98 (0.78–1.24)	1.00	0.38
Frail	1.17 (0.69–1.99)	1.37 (0.89–2.11)	1.00 (ref)	1.56 (1.13–2.13)	1.62 (1.22–2.16)	0.01	0.75
			Diastolic blood pressure, mmHg				
	<60	60–69	70–79	80–89	≥90		
			OR** (95% CI)				
Non–frail	1.34 (0.84–2.13)	1.30 (1.01–1.67)	1.00 (ref)	1.05 (0.85–1.28)	1.00 (0.78–1.27)	0.09	0.28
Frail	0.82 (0.45–1.49)	1.03 (0.75–1.40)	1.00 (ref)	1.05 (0.82–1.33)	0.97 (0.72–1.30)	0.89	0.49

*Non-frail: FI < 0.25; Frail: FI ≥ 0.25.
**Adjusted for age, sex, education status, current smoking and drinking status, marriage status, education years and body mass index.
Abbreviations: OR: Odds ratio; CI: Confidence interval.

Supplementary Table 6. Odds ratios for severe falls associated with systolic blood pressure and diastolic blood pressure, stratified by frail status.

Frail status*	Systolic blood pressure, mmHg					p-trend	
	<110	110–119	120–129	130–139	≥140	linear	quadratic
			OR** (95% CI)				
Non–frail	1.06 (0.58–1.93)	0.65 (0.37–1.14)	1.00 (ref)	0.79 (0.53–1.18)	0.81 (0.57–1.14)	0.55	0.86
Frail	1.45 (0.69–3.07)	1.20 (0.62–2.33)	1.00 (ref)	1.54 (0.95–2.50)	1.51 (0.97–2.35)	0.25	0.66
			Diastolic blood pressure, mmHg				
	<60	60–69	70–79	80–89	≥90		
			OR** (95% CI)				
Non–frailty	1.32 (0.66–2.66)	1.07 (0.71–1.60)	1.00 (ref)	0.89 (0.64–1.24)	1.17 (0.81–1.68)	0.93	0.14
frailty	0.80 (0.33–1.96)	0.81 (0.50–1.31)	1.00 (ref)	1.05 (0.73–1.50)	0.89 (0.57–1.39)	0.64	0.30

*Non-frail: FI < 0.25; Frail: FI ≥ 0.25
**Adjusted for age, sex, education status, current smoking and drinking status, marriage status, education years and body mass index.
Abbreviations: OR: Odds ratio; CI: Confidence interval.
